# Reports on Hospitals of the United Kingdom

**Published:** 1914-05-02

**Authors:** Henry Burdett


### REPORTS ON

### Hospitals of the United Kingdom.

By SIR HENRY BURDETT, K.C.B., K.C.V.O.

### SERIES III.

### RAMSGATE GENERAL HOSPITAL AND SEAMEN'S INFIRMARY.

This institution was established in 1848. The new buildings were erected in 1909, the cost being defrayed out of a legacy of £16,000. The hospital occupies an ample site with abundant garden space where all the vegetables used in the hospital, except potatoes, are grown. It contains twenty-six beds and has an excellent operation theatre, which, however, is defective because the architect has not fulfilled the requirements of an operation unit. The large steriliser, for example, is kept outside in the corridor. The sanitary blocks, cross-ventilated passageways, and some of the fittings are good, but other fittings, owing to the patterns of two different manufacturers being fixed, are not satisfactory. The ward floors and a few others are of Canadian white willow wood, polished. They presented a pleasing appearance and are to be commended to hospital managers generally, Canadian white willow being infinitely preferable for ward floors to some woods used in the past, which have been found a failure. The larger wards have verandahs where the patients can sleep out at night. We have found in the course of our inspections a growing tendency on the part of the resident medical staff to sleep in the open air. The present house surgeon at Ramsgate follows this practice.

### Cupboards and Decoration.

This hospital is better provided with linen cupboards of a good pattern than most institutions of its type, though these and other cupboards require better ventilation. The whole of the linen and bedding is good with the exception of some flock mattresses and pillows, which should be scrapped at once.

On the whole, the hospital presents an attractive appearance outside, especially as it has recently been redecorated. The inside of the hospital needs renovation. The committee would be well advised to organise a definite scheme for the whole building so as to secure that the wards and other portions of the hospital are recoloured and redecorated throughout, at least once in two years. There is an entirely new mortuary unit, enclosed in a fence, with ample accommodation, but the details have never been carefully thought out, and there are several small matters which should receive the immediate attention they deserve.

Unfortunately, the accounts are not kept on the Uniform System, as they ought to be, and it is not easy to arrive at the actual financial position for the year. For the same reason no comparison can be made between the cost of this hospital and others of similar size, seeing that the statement of accounts is so muddled and unclassified as to render any analysis, or an attempt to get the expenditure under the proper heads, practically impossible. This is a matter which we commend to the personal attention of the Chairman, Captain Marling, together with others which we shall mention later. On the face of the accounts a deficit of £410 for the year is declared, but as this includes on the expenditure side £180 for a legacy carried to the cash account, commemorative tablets, certain buildings, and various other matters which until classified cannot be dealt with as ordinary expenditure, we incline to the feeling that the probability is that the deficiency, if any, does not exceed one half of the amount mentioned in the accounts. Bad accounting often means careless expenditure, and the business side of the affairs of this hospital, if we may judge by the inchoate manner in which the statement of accounts is presented, seems to call loudly for reform and the adoption of the Uniform System of Accounts on and from February 1, 1914. It is impossible from the way the income and expenditure accounts are set forth and the unnecessary repetition of details, without classification, which the accounts exhibit, to ascertain accurately any complete figures. Judging by the relatively small sum obtained from subscriptions—namely, £382—and the fact that Ramsgate has a population of 30,000 or more, it should not be difficult to increase the income of this hospital to any amount necessary to maintain it in the highest state of efficiency.

### A Chance for an Administrator.

Speaking generally, we should say that the hospital still exhibits evidence of its comparative newness. It will be improved when someone in authority goes exhaustively through the whole establishment, looks carefully into the arrangements of the lavatories, ward kitchens, linen cupboards, storerooms, and other portions of the buildings, and decides where the ventilation and many details of the furnishing can be improved. Then the Chairman should secure that all these improvements are effected without delay. We may instance the nurses' sitting room at this hospital as typical of what we have in mind. It presents the appearance of a board or business room at its worst. It contains some high-backed, elaborately uncomfortable chairs, which are utterly out of place in a nurses' sitting room, for it ought to be comfortably restful and attractive for recreation. Few or none of the nursing staff when off duty, we imagine, ever use this extraordinary example of what a nurse's recreation room ought not to be.

